# It takes two to tango: A directed two-mode network approach to desirability on a mobile dating app

**DOI:** 10.1371/journal.pone.0327477

**Published:** 2025-07-23

**Authors:** Renata Topinkova, Tomas Diviak

**Affiliations:** 1 Department of Sociology, LMU Munich, Munich, Germany; 2 Department of Sociology, Faculty of Arts, Charles University, Prague, Czechia; 3 Institute of Sociology, The Czech Academy of Sciences, Prague, Czechia; 4 Department of Criminology and Mitchell Centre for Social Networks Analysis, University of Manchester, Manchester, United Kingdom; Duke University, UNITED STATES OF AMERICA

## Abstract

Using digital traces from online dating presents the opportunity to study the earliest stages of human mating. We focus on whether online dating app users are homophilic in terms of the desirability of whom they pursue. Using data from a Czech online dating app, we construct directed two-mode networks where nodes represent users, ties represent messages expressing interest (“swipes”), and desirability is measured by the number of “swipes” each node receives. Using network measures and conditional uniform graph tests extended to directed two-mode networks, we find that the structure of the networks is considerably hierarchical. Women are in advantageous position on the app due to the uneven gender ratio and their substantially higher desirability. The results further show that men pursue women who are more desirable than themselves. The reciprocated contacts are comparatively more homophilic. These results suggest that in terms of desirability, the similarity of partners is due to the subsequent mating processes (e.g., rejection) rather than due to initial preference for similarity.

## Introduction

Online dating is the fastest-growing way for couples to meet. For instance, recent studies in the US find that almost one-third of newlyweds met online [1]. The percentage of long-term couples that originated online steadily rises, with the most recent studies reporting almost 40% of new heterosexual couples meeting online, making it the most frequent way to meet a romantic partner [2]. Thus, it is safe to assume that online dating is no longer marginal and is here to stay. Even though more and more couples meet online, the conditions and mechanisms of how it happens are still not completely understood. One of the persistent patterns in partnership formation research is homophily, that is, the fact that the partners tend to resemble each other in terms of various characteristics [3]. While there are studies investigating the effect of online dating on educational homogamy [4,5], racial homophily [6–9], or homophily on political ideology [10], these come mostly from the United States or Western Europe, and it is unclear whether these results hold in different contexts [11].

Online dating presents us not only with a new way of meeting partners, but also with entirely new data that allows us to examine the search for a partner in its earliest stages, including the initial trials and failures in forming an intimate partnership. Exploring these initial stages of searching for a partner is usually not possible in a traditional survey design. Using this data gives us access to a large number of individuals interested in pursuing intimate relationships regardless of their relationship status; it allows us to observe users’ behavior rather than their stated preferences; and it provides us with information regarding the number and characteristics of the available romantic partners.

Our study makes use of digital trace data from a Czech online dating app from July 2017 to examine the patterns of homophily on desirability, i.e., their attractiveness in the context of the online dating app. In particular, we aim to answer the following questions: Is there a hierarchy of users in the online dating market? How does the position of women and men differ in its structure? Do users contact similarly desirable counterparts, or do they aim for more desirable ones? Are the successful matches more often between similar or dissimilar users? Beyond answering these research questions, our study contributes to the research on online dating by examining the structure of an online dating market and its underlying mechanisms (preference, structure, and non-reciprocity/competition) with regard to homophily on desirability [12] in a Central European area, namely Czechia.

We examine users’ search patterns in two major cities in Czechia (Prague and Brno) using social network analysis (SNA; [13,14]). SNA is an approach that focuses explicitly on the structure of networks emerging from interactions among individuals and their positions within these networks. Thus, SNA allows us to assess the dating market structure, identify the desirable actors, examine who contacts them, and whom they contact. The interaction between users on online dating sites, i.e., sending and receiving swipes (expressions of interest on the platform), gives rise to a network structure of the online dating market [12]. Within this network, some users receive more swipes than others, which results in a hierarchy of users’ desirability.

From a methodological point of view, heterosexual online dating markets present a challenge for SNA. Users of the app in this context are allowed to swipe (i.e., send ties) only to users of the opposite gender, not to those of the same gender. In SNA, this would give rise to a two-mode or bipartite network in which ties are permitted only between modes (distinct classes of nodes, typically individuals and their affiliations), but not within them [15,16]. However, two-mode networks are universally considered to be undirected, that is ties are conceptualized as inherently mutual. Yet, in online dating apps, it is not only possible, but theoretically important to consider the direction of the ties (swipes) as it allows us to distinguish who is interested in whom and even more importantly, whether this interest is reciprocal (a ‘match’ in online dating terms). In order to properly analyze the directed two-mode networks in our data, we therefore extend existing methods for two-mode networks to account for direction of ties. To our knowledge, our study is the first one to do so in a social-scientific context, making it innovative both substantively and methodologically.

## Theoretical background

Homophily is the tendency to associate with individuals who are similar to us [3]. If the end point of this process translates into a marriage, we refer to it as homogamy. Indeed, decades of research show that spouses often share the same level of education [17,18], status [17], ethnicity [19], or age [20]. These patterns were also observed in dating or cohabitating couples [21], and initial stages of online dating, for instance, in case of ethnicity [9,22], smoking or other lifestyle choices [23,24].

The origin of homogamy is usually explained by either individual preference or by structural constraints [3,25]. First, individuals prefer to match with someone like them, as they may have more in common and share interests, which in turn makes it easier to fall in love (matching hypothesis, [26]). Second, the structural constraints of the real world such as geographical proximity, social distance, or educational system make similar individuals more likely to meet [e.g., 27,28]. Despite the importance of those two mechanisms in mate choice, data on the opportunity structure are rarely available, and thus it remains difficult to disentangle the two mechanisms [29].

However, formed unions do not always correspond to individuals’ initial preferences or structural constraints. For example, even though we observe age homogamy, with the most common age difference between spouses being one year in Czechia [20], data from online dating research clearly show that this is neither the initial preference of individuals nor the result of structural constraints imposed on them [30]. This brings us to another explanation of homogamy origins through competition [11] or non-reciprocity [31]. Even though individuals may prefer someone with a higher market value than themselves (i.e., younger or more attractive), if neither partner is willing to partner down, the resulting couples will be homophilic regardless of the initial preference [11]. Schaefer [31] views non-reciprocity as an ongoing exchange, in which heterogenous couples are more likely to dissolve with each additional exchange between partners (e.g., additional messages between online daters). The mechanisms of competition or non-reciprocity can be especially pronounced in the online dating environment, as the costs of approaching someone more desirable than oneself can be lower than offline, and the expansion of one’s dating pool also brings the expansion of one’s competition.

The online environment modifies some aspects of dating, which in turn affect the behavior of users in their mating pursuits online. The first of the distinct features of online dating is that it weakens the structural constraints. In more traditional settings, one can only physically encounter a limited number of prospective partners. In contrast, online dating significantly widens the dating pool both in size and variety. Since it does not require singles to share the same physical space or have mutual acquaintances, there is a possibility of encountering and matching with a more dissimilar mate than in more traditional meeting ways [32,33].

The second specific aspect of online dating is that to express interest online, users send each other messages or indicate their interest through specific features of a particular dating site, such as likes or swipes. In comparison to real-life settings, expressing interest online requires fewer resources in terms of time and money to approach a prospective mate. This is especially true in mobile dating apps that enable dating anytime and anywhere. Furthermore, it may be less intimidating to approach a prospective mate online as it does not require face-to-face interaction at the time of the first message, eliminating the potential social stigma of rejection and decreasing the impact of negative emotions related to rejection face-to-face [33]. Lastly, online dating eliminates rejection due to the unavailability of mates, as everyone who joins an online dating site is presumably looking for some kind of relationship, regardless of their actual relationship status. The lower costs of initiating a contact online can translate into users being more ambitious while pursuing a partner than they would have been in a more traditional setting.

By engaging in online dating, users not only send but also receive swipes or other expressions of interest (e.g., messages) from others. The number of swipes users receive reflects their overall desirability on the dating market. These messages may give rise to a hierarchy among users as some receive many swipes, indicating their high desirability, while others receive a low number of swipes, indicating low desirability. Indeed, previous research shows that the distribution of received contacts is skewed: a few users receive a disproportionate amount of interest, while most users receive only a few [34]. It is difficult to say exactly what constitutes being desirable online, but it is safe to assume it reflects various underlying characteristics, such as the user’s age, status, attractiveness or other attributes, that can be either explicitly provided by users in their profile text or pictures or inferred by others through viewing their profiles. Studies show that similar hierarchy exists in offline mating pursuits; however, one could argue that such hierarchy is even more pronounced online [12] due to the large pool of available partners and the low costs of sending additional messages.

Although users typically cannot observe such hierarchy directly and do not know how many messages others receive, research shows they can assess their desirability and act accordingly [12,35]. Users have been shown to be relatively good judges of the desirability of others and adapt their behavior when they perceive the prospective partner to be highly desirable, such as by engaging in a higher level of deception [36] or sending longer messages [12]. Most importantly, users can directly evaluate their desirability based on the interest (i.e., messages, swipes) they receive and the response rate from users they have pursued.

In this paper, we are using the term “desirability” instead of “attractiveness”. While the physical attractiveness is undoubtedly a significant part of users’ desirability, other factors likely also play important role: for instance, age, education, and the overall profile presentation and quality (e.g., selection of photos, lifestyle choices, social status cues, text in short biography).

### Structure of the dating market

In this section, we formulate research questions based on what is known about dating markets in general, what we outlined as specific for online dating, and how it translates into the structure of online dating. Note that when we observe the similarity of existing couples, we observe the end product of a long and often tedious mating process. In the initial stages, individuals may make many unsuccessful attempts that remain hidden while examining the already formed unions such as married couples. Therefore, we focus not only on the successful matches but also on the unsuccessful attempts.

Previous research shows that there is a variance in the number of likes that online dating users receive [e.g., 12,34,37]. This variance results in a hierarchy among users and that users display some awareness of this hierarchy and their position within it [12,35]. The position of each user within the hierarchy has a profound effect on how they experience online dating. While those at the top of the hierarchy receive many likes and may choose from a large pool of available partners, users at the bottom may find very little or even no matches [34]. For this reason, we first want to verify whether the dating app in our data displays a hierarchical structure or not. If the structure is hierarchical, it should exhibit considerable variance in the desirability among the users, with a few highly desirable users attracting a vast number of swipes and a relatively high number of users receiving only a few swipes. Therefore, we formulate the first research question as follows:

RQ1: *Is there a hierarchy in desirability among the users?*

Related to the hierarchy of users’ desirability, there is ample evidence that women enjoy a better position on online dating sites than men. Nearly every dating site and app reports a skewed men-to-women ratio, with men outnumbering women significantly [34,37,38]. The lower proportion of female users puts women inherently under more interest from the more numerous men, placing them higher in the hierarchy. It also appears that there is a prevailing gender norm in terms of who is expected to contact whom, placing the burden of the first move on men as it frequently is offline [39,40]. In terms of violating this gendered expectation online, there is mixed evidence: Kreager et al. [37] show that women who initiate the first contact are rewarded with higher-value partners, while Dinh et al. [41] show these attempts are penalized. Since the experience of online dating is quite different for men and women, they may also employ different strategies in their pursuits. In line with this reasoning, all the subsequent research questions examine men and women separately.

RQ2: *Are there differences between men and women regarding their desirability (average number of swipes)?*

Previous research on online dating shows that users seek similarity in some characteristics, such as education or ethnicity [23,24], but aspire to maximize on others, such as attractiveness [34]. In line with this, studies show that initial messages in online dating are not directed towards similarly desirable mates but more often towards higher-value mates [12,37]. This phenomenon is referred to as aspirational pursuit [12], vertical preferences [23], or the mechanism of competition [18,29]. As stated earlier, the aspirational pursuit may be more pronounced in online dating due to the lower costs of contacting a prospective mate. In the network, this would be manifested as a large difference between the desirability of the pursuer (sender of the swipe) and the pursued (receiver of the swipe).

RQ3: *Do users exhibit aspirational pursuit or preference for similarity?*

Although preference and aspirational pursuit are two very different mechanisms, they can generate the same outcome: homophily. This paradox can be explained by the mechanism of non-reciprocity [31]. Non-reciprocity refers to a situation in which individuals desire relationships with more desirable counterparts. However, these counterparts, themselves aiming for more desirable partners, reject those attempts while being rejected by those more desirable than them. According to Schaefer [31], the exchange of being repeatedly rejected and repeatedly rejecting ultimately leaves individuals to pair off with similarly desirable mates which aggregates into similarity at the level of the whole market. Kreager et al. [37] confirm that dissimilar matches are more likely to dissolve, and thus the resulting pairs are more similar. The reciprocated contacts, that is contacts between mutually attracted users, should therefore be more homophilic in their desirability. In other words, users in the reciprocated mutually interested pairs would have similar amounts of received swipes.

RQ4: *Are the successful (reciprocated) attempts more often homophilics?*

## Data

We use data from a Czech online mobile dating app operating between 2016 and 2019. The data was obtained upon an agreement with the app providers in an anonymized form. It includes 10,528 users who were active in July 2017. Each user is represented by a unique ID. Only the first swipes and the responses to them, if any, were collected. We focus solely on heterosexual searches, as there were not sufficient data on same-sex search patterns. We only include active users in the analysis, defined as users who sent or received at least one swipe during the collection period. According to the app provider, the app was downloaded over 50 000 times at the time of data collection. Downloading and using the app was free, although it did offer a paid membership. However, out of more than 10 000 users who were actively using it in July 2017, less than 100 users had a paid membership. The app primarily attracted young, highly educated Czechs living in big cities (mean age 28; 64% men).

At the time of data collection in July 2017, the app’s main purpose was to eliminate long exchanges of messages and quickly facilitate meetings offline instead. To do so, it presented users in the closest geographical proximity. As far as we know, there was no other, more sophisticated algorithm that matches users (e.g., desirability score, past choices). Here, we rely on the information we received from the app providers. The reason for the lack of a more sophisticated algorithm is its purpose of facilitating offline dates as quickly as possible, and that the app was relatively new at the time. The app showed one user at a time, forcing users to make a decision about each presented user sequentially. If interested, users could swipe a “Date now” button, which subsequently sent the other user a notification that someone was interested in them. These notifications were shown one at a time forcing users to make a decision about each user to see other notifications they have received. Moreover, users had to decide within 24hours whether they are interested or not. If they chose to accept, a chat window opened where users could communicate with their chosen counterpart. Users could not message each other before accepting the invitation. Unlike on the well-known dating app Tinder, swiping is not double-blind, meaning that if one party swipes the other, the pursued party is immediately notified of the fact. Therefore, unlike on Tinder, users receive immediate feedback on their desirability as every time someone likes them, they receive a notification and an opportunity to reciprocate.

We selected data from two of the major cities, Prague (n = 2,321) and Brno (n = 624), for the analyses. The reason for selecting the two cities were twofold: first, they are the two most populated cities in the country as well as in the dataset; second, it is reasonable to assume that each city is its own dating market within which users have a real chance to meet in person, and thus be shown to each other by the app. Thus, even though we lack the information about which profiles a user has seen, narrowing the analysis down to cities combined with the proximity-based algorithm reduces the pool of available partners to those the users could have potentially seen and could eventually meet in person.

One of the main advantages of using data from online dating is that it allows us to observe users’ actual behavior online. This way, we can observe the initial stage of online dating, including the part that is inevitable, yet usually hidden in more traditional types of research: initial rejection. Moreover, since users are not aware that they are being observed, they can express their preferences more freely. A vast majority of studies on partner preferences has been done via surveys which may be prone to social desirability (e.g., individuals may not want to admit who they contact, or how often they get rejected), assume that stated preferences translate into actual behavior, or are otherwise affected by problems with recollection or simply by not reflecting one’s true preferences. Other studies focus on explaining the similarities between spouses. It is, however, difficult to disentangle whether couples got together as a result of their similarity, or they became more similar over time (“adaptive socialization” [42]). Additionally, unlike other data sources, online dating provides us also with a snapshot of the opportunity structure of available partners [29].

Despite these advantages, utilizing online dating data has several limitations. First, this data is ready-made. Ready-made means that the data was not collected for the purpose of academic research but rather as a byproduct of the app [43]. Consequentially, the data only provides limited information about users. In our case, we have information about users’ gender, age, location, whom they contacted, and whether this contact was reciprocated (accepted) or not. Thus, we lack any information regarding other sociodemographic variables (e.g., education, income), technical information about profiles (e.g., date of registration on the app, time spent using it), motivations or whether the matches met offline or not. While we have the information about users’ age, we do not include it in further analyses as it has been analyzed in previous research based on this dataset [30].

Second, there is always the possibility of algorithmic confounding while working with online generated data [43]. Algorithmic confounding refers to the fact that websites and apps follow their own goals (e.g., financial gain), which influences users’ behavior on the app, and can thus introduce patterns that would not have been observed otherwise [43]. In turn, data obtained from these sources may reflect not only users’ true behavior but also the technical settings of the given app such as its graphical layout or the way the information is presented to users. As we stated above, the only operating algorithm on the app was matching the users of geographical proximity, which we account for by only selecting users from two major Czech cities (Prague and Brno) and analyzing those two cities separately.

Third, bots are ubiquitous on online dating. Bots are “automated third-party programs trying to make users engage into contact and eventually into an over-priced and useless external product” [44]. In online dating, bots typically pose as attractive young women trying to lure men outside the app, where they then solicit money [44], and can account for a large part of the traffic on online dating sites. As such, they can distort the sociodemographic distribution and induce artificial behavioral patterns – for example, reducing the selectivity of attractive young women. We took several measures to limit the bias that could be introduced by bots. We tried to identify possible bots in the following way: we filtered out women who accepted (reciprocated) all the swipes they received, given that they have received at least 30 messages. We chose this criterion because there may be women who accepted all swipes because they had received only a few of them, and because women who received a lot of swipes do not have to be necessarily bots, they could only be highly desirable. After identifying such users, we omitted them from further analysis (Prague: n = 49, Brno: n = 5).

## Methods

To answer our research questions, we use methods from social network analysis (SNA; further see [13,14]). SNA is concerned with analyzing networks consisting of nodes and ties among them. In our case, the nodes represent the users of the dating app, and the ties represent the swipes users send to others to express their interest. Because our data contains only the heterosexual part of the dating market, there can be no same gender swipes. This means our data forms directed two-mode networks. In a two-mode network, there are two distinct classes of nodes (modes, in our case men and women) with ties permitted only across the modes, not within them, insuring no same gender swipes. Directed two-mode networks are a special class of networks for which standard methods for two-mode networks cannot be readily applied. For this reason, we had to modify our measures.

A standard two-mode network is captured in an incidence matrix with dimension *n* *×* *m*, where *n* refers to the number of nodes in the first mode while *m* to the number of nodes in the second mode with entries a_ij_ = 1 denoting that node *i* is affiliated with node *j*. Such incidence matrix is not sufficient for capturing directed two-mode networks though, as it can only store ties in one direction (*i *➔ *j*or *j *➔ *i*, but not both). For this reason, we use two incidence matrices instead – one for each direction. Thus, we have a female sender matrix M_F_ with dimensions *n*_*females*_* × n*_*males*_ and entries a_ij_ = 1 denoting that women *i* sends a tie to male *j*, analogously for the second male sender matrix M_M_ with dimensions *n*_*males*_* × n*_*females*_.

Indegree and outdegree refer to the number of ties a node has received or sent respectively. Indegree of women is calculated from the male sender matrix as column sums, whereas outdegree of women is calculated from the female sender matrix as row sums. This is calculated analogously for men. Indegree is our measure of desirability as it captures how many users are interested in a given user, while outdegree refers to the activity of each user. Since there are different numbers of men and women in each network, indegrees and outdegrees had to be standardized for the calculations that require comparability between men and women. The standardization was done by dividing the in/outdegree by the maximum possible, that is, by the number of users of the opposite gender on the platform. For instance, if there is a woman with standardized indegree of 0.5, it means that she received swipes from exactly half of the male users.

To describe the entire structure of the networks and the extent to which they might be hierarchical, we used average, standard deviation, and skewness of in/outdegrees together with density, indegree centralization, and reciprocity. Large standard deviations of indegrees relative to their average and their positive skewness indicate focus of swipes on a handful of particularly desirable users. Density is the ratio of the observed ties to all the possible ties. Compared to traditional two-mode networks, we also must account for direction of ties. Therefore, we calculate density as follows:


$$Density=\frac{{\mathrm{\backslash sumM}}_{F}\;+\;{\mathrm{\backslash sumM}}_{M}}{n_{females}\;*\;n_{males\;}*\;2}$$

For instance, a density of 0.1 would indicate that 10% of all possible ties are present in the network. Indegree (desirability) centralization denotes the ratio of the observed dispersion of ties to their maximum possible dispersion and it was separately calculated for men and women based on Freeman’s [45] approach. The observed dispersion is calculated as the sum of differences in indegrees between the observed most central node and the remaining ones, while the maximum possible dispersion is calculated as this sum among the same number of nodes and ties in which all the ties are directed towards a single node (a star graph). Formally:


$$Centralization=\frac{\sum_{i=1}^{n}\left(\max\left({indegree}_{observed}\right)-\;{indegree}_{i}\right)}{\sum_{i=1}^{n}\left(\max\left({indegree}_{star}\right)-\;{indegree}_{i,star}\right)}$$

Consequently, a centralization of for instance 0.3 would mean that the ties in the network are centralized to 30% from their hypothetical maximum centralization. Thus, indegree centralization higher than expected by random chance indicates the presence of hierarchy in line with RQ1. Lastly, reciprocity is the ratio of reciprocated ties (i.e., ties that have their counterpart in the opposite direction) to all the ties in the network:


$$Reciprocity=\frac{2*{\mathrm{\backslash sumM}}_{R}}{{\mathrm{\backslash sumM}}_{F}\;+\;{\mathrm{\backslash sumM}}_{M}}$$

Where M_R_ is the matrix containing only the reciprocal ties (mutual swipes, i.e., the a_ij_ in M_R_ = 1 if a_ij_ in M_F_ and a_ji_ in M_M_ are both equal to 1). For instance, a reciprocity of 0.25 means that a quarter of all ties are reciprocated, that is, a quarter of all swipes lead to a match.

To answer our four research questions, we used non-parametric randomization-based methods, because we do not study a sample from a population, but rather the entire population of the dating app users in the two cities. Thus, our inference does not aim to generalize to an underlying population, but rather to infer whether our results are likely to arise by random chance under given certain circumstances or not. This is a common scenario in SNA [cf. 13,14,45]. For RQ2 about the differences in desirability between men and women, we used a two-sample randomization test comparing standardized indegrees of both genders. For the remaining two research questions, we calculated the difference between the standardized indegrees of senders and receivers of all ties (RQ3), and the difference between the standardized indegrees of senders and receivers of reciprocated ties only (RQ4). In order to test whether the given difference could be equal to zero, and thus could have possibly arisen by chance, we developed a conditional uniform graph (CUG) test [46,47] for our directed two-mode networks. We simulated a benchmark distribution of 1,000 alternative networks in which senders chose different receivers for their swipes by randomly permuting the entries in the rows of both M_F_ and M_M_. This preserves the overall number of swipes and the row totals (outdegrees), but changes indegrees in each matrix, changing who the user sends swipes to but not changing the total number of the swipes they send. For each such permuted matrix, we calculate the difference between the standardized indegrees of senders and receivers. This procedure yields a distribution of possible outcomes in terms of networks that are similar to our empirical ones while conditioning on their size and outdegree distribution (and thus on density as well). This distribution enables us to calculate the empirical p-value which is the proportion of the outcomes in the distribution that have the same or more extreme values than the observed value [47]. As a sensitivity analysis, we adjusted (i.e., weighted) the swipes so that swipes from highly active users weight less than those from less active ones. To achieve that, we created new matrices of sent ties where the ties are valued as a fraction of each sender’s total number of sent swipes (outdegree). We then conducted the same CUG tests as described above.

For testing RQ4 that focuses on reciprocated ties only, we simultaneously permuted both M_F_ and M_M_ and for each pair of these permuted matrices, we generated a new reciprocal matrix M_Ri_. Subsequently, for each M_Ri_ we calculate the reciprocity and the differences between each pair of nodes incident on every tie. We then calculated the empirical p-values as described above. Subsequently, we compare the desirability gap between the senders and the receivers of swipes regardless of their success, and the desirability gap between the senders and receivers of the mutual interested users (reciprocal matches).

All analyses were performed using R Statistical Software [48]. The reproduction package is available at OSF (https://osf.io/6zcrs). The study was approved by the ethics committee of The Institute of Sociology of the Czech Academy of Sciences under number SOÚ-148/2025.

## Results

### Network structure

We first describe the structure of the two dating networks in our study. In the Brno case, there are 624 users (20.4% of which are women) with 5,260 swipes among them, whereas the Prague network is almost four times as large with 2,321 users (24.9% women) and 36,665 swipes among them. On average, users receive and send 8.43 swipes from others in the Brno network and 15.8 in Prague (Table 1). Indegrees, our measure of desirability, displays a very high variability indicated by its standard deviation of 24.01 and skewness of 5 in Brno. In the Prague network, the standard deviation of indegrees is 45.56 and skewness 5.23, suggesting that some users may even have triple the average amount of received swipes in both networks. The maximum indegree in the Brno network is 204 received swipes, while some of the users in Prague received as much as 418 swipes. The distribution of desirability in both markets is strongly positively skewed with a few highly desirable users receiving a disproportionate number of swipes and a lot of users receiving none or almost none. In terms of outdegrees (sent swipes), we can see certain variance across users with a standard deviation of 8.79 and 18.79 in Brno and Prague respectively. This means that on the one hand some users sent almost no swipes, whereas on the other hand some other users were highly active in searching for a partner.

**Table 1:** Descriptive statistics for Brno and Prague networks in terms of age, indegree and outdegree.

*Brno*	Mean	SD	Median	Skewness
Age	28.14	6.75	27	0.99
Indegree	8.43	24.01	1	5
Men	1.58	2.27	1	3.17
Women	35.23	43.83	18	2.01
Outdegree	8.43	8.79	5	1.45
Men	9	8.98	6	1.44
Women	6.19	7.64	3	1.4
*Prague*	Mean	SD	Median	Skewness
Age	28.93	6.52	28	0.75
Indegree	15.8	42.56	3	5.23
Men	4.22	6.16	22.5	3.17
Women	52.71	75.41	2	2.37
Outdegree	15.8	18.79	9	2.34
Men	16.53	18.18	10	1.8
Women	13.47	20.45	7	3.66

The density of the network is 0.04 in the Brno case and 0.02 in the Prague case, indicating that about 4% or 2% respectively of all possible ties are present in the network (Table 2). We also calculated the indegree centralization separately for men and for women in both networks. In the Brno network, the value of 0.15 for men suggests the incoming swipes are centralized for about 15% of their maximum possible centralization, while this reaches 34% for women. This is similar in the Prague network with indegree centralization among women far higher (21% of the maximum) than among men (11%), pointing at substantially steeper hierarchy among women than among men as the differences in the number of incoming swipes is much more pronounced among women in both networks. The values of centralizations are for both networks and both genders substantially higher than in any of the simulated networks indicating that such centralization is much higher than we would expect by random chance given the size and outdegree distribution of the networks. Lastly, the reciprocity in the Brno network is 0.27 indicating that in a bit over one quarter of the swipes the interest is mutual, whereas in Prague almost two fifths (38%) of all swipes are reciprocated.

**Table 2:** Whole-network measures describing structures of Brno and Prague networks.

	Brno	Prague
Density	0.042	0.019
Indegree centralization		
Men	0.15	0.11
Women	0.34	0.21
Reciprocity	0.27	0.38

Overall, these results suggest that the dating networks are considerably hierarchically structured in terms of desirability with large differences among users and with the hierarchy being more pronounced among women and thus provide a positive response to RQ1.

### Received swipes comparison of men and women

**Fig 1:**
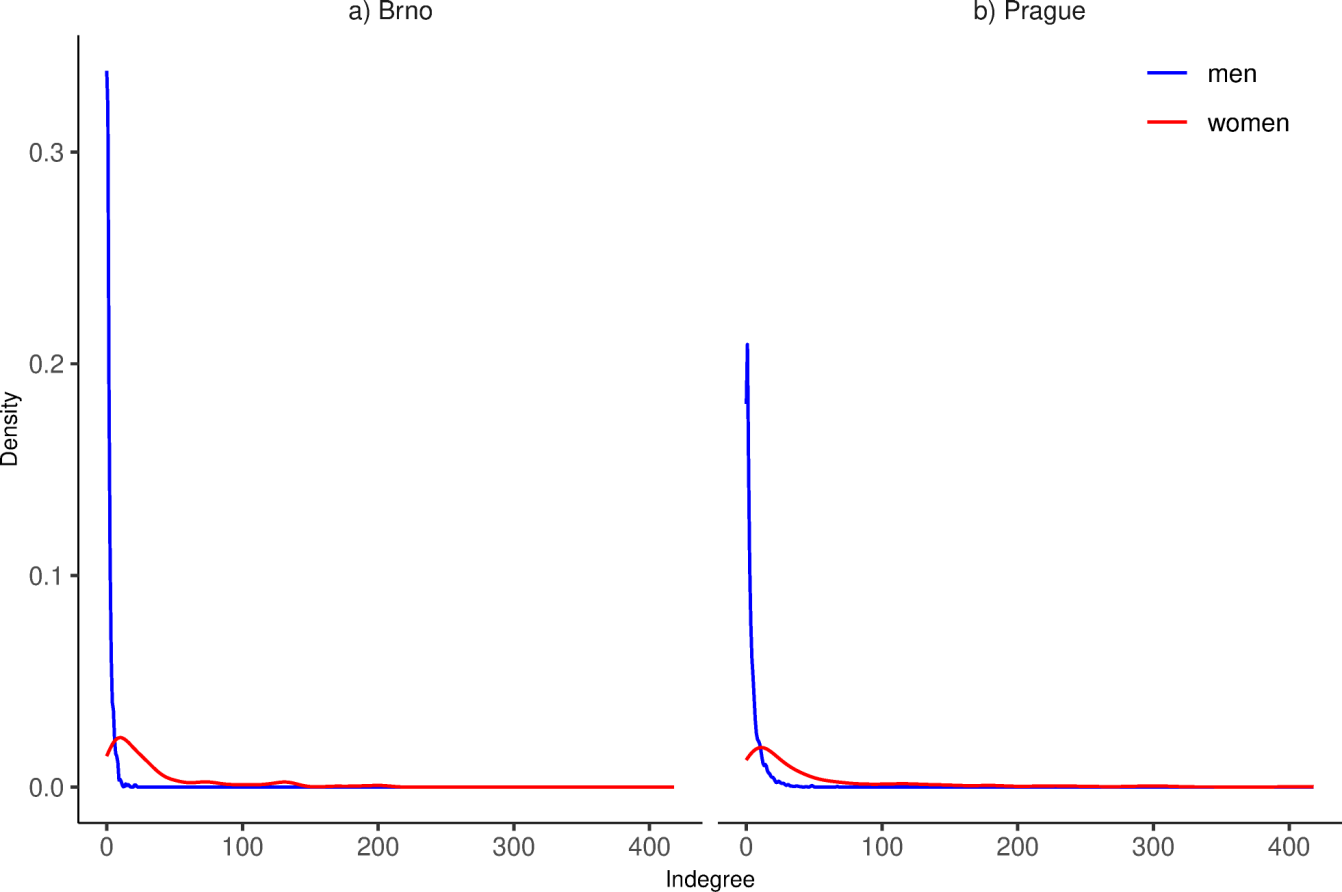
Density plots of received swipes (indegree). Men (blue), women (red).

Fig 1 displays density plots comparing the desirability (unstandardized indegree) of men and women in the two markets. Both the markets display a similar pattern that is in line with previous research on online dating [e.g., 34]: while there is a considerable heterogeneity within both genders in terms of their desirability, women receive far more swipes than their male counterparts. Vast majority of men (even the highly desirable ones) receive less swipes than women with below average desirability. This difference holds even for the indegrees standardized by the number of users of opposite gender in the given area. The Cohen’s d value for the comparison of men and women on their standardized indegree is 1.37 in Brno and 0.97 in Prague, suggesting a difference of about one overall standard deviation between the genders in both markets. Therefore, the response to RQ2 is that there are high differences between the desirability of men and women in our data in both locations. Women are thus in a better position than men, as they receive substantially more swipes putting them in the “choosing” position. This can be caused by the skewed ratio of men and women since the scarcity of women on the app makes them automatically more desirable and some women may be flooded with attention.

### Aspirational pursuit

Table 3 gives an answer to RQ3 of whether there is an aspirational pursuit. The aspirational pursuit would be manifested by users contacting prospective mates who are more desirable, i.e., by a negative difference between senders’ and receivers’ standardized indegrees. In the Brno network, this difference is on average 0.03 for ties send by women, indicating that women tend to nominate on average slightly less desirable mates (by about 3% of the possible maximum). The situation is different for ties sent by male users, because the difference there is −0.16, indicating that men tend to send ties to women who are on average considerably more desirable than themselves. Both these figures can be seen as salient patterns that cannot be attributed to random chance, given the size and outdegree distribution in the network, as it is seen from the empirical p-values showing that there is no network in the simulated distribution with the same or larger differences between the standardized indegrees of senders and receivers. In the Prague network, the situation is rather similar with the difference for ties sent by women being 0.02, while reaching −0.08 for ties sent by men indicating slight dating “down” tendencies for women and aspirational tendencies among men. The empirical p-values again show that these results are highly unlikely to arise by random chance given the network size and outdegree distribution. All these findings together point toward the presence of aspirational pursuit among men, but not among women.

**Table 3:** Conditional uniform graph tests results. Each row represents the given network with its observed mean together with its simulated mean, standard deviations, and empirical p-values based on 1,000 permutations of the network.

	obs. mean	Pr(<=obs. value)	Pr(>=obs. value)	sim. mean	sim. SD
Brno					
Men contact women	−0.157	0.00	1.00	−0.050	0.0002
Women contact men	0.031	0.00	1.00	0.049	0.0005
Reciprocal	−0.032	1.00	0.00	−0.053	0.002
Prague					
Men contact women	−0.075	0.00	1.00	−0.015	0.0003
Women contact men	0.020	0.00	1.00	0.034	0.0001
Reciprocal	−0.019	1.00	0.00	−0.021	0.004

To ensure that our results are robust, we conducted a sensitivity analysis. We adjusted the swipes by senders’ outdegree so that swipes from indiscriminately swiping users count less than those from more selective ones. The results are overall the same as the results from the main analysis: women nominate on average slightly less desirable mates, and men send ties to women who are on average considerably more desirable than themselves. The results are highly unlikely to arise by random chance given the network size and outdegree distribution. We report the full results of the sensitivity analysis in S1 Table. Thus, we respond to RQ3 by stating that there is aspirational pursuit on desirability among men in both locations.

### Homophily of reciprocated swipes

To answer RQ4, we filtered out only the mutually attracted pairs of users from both networks, that is pairs with reciprocated ties. Table 3 shows the results in the same way as for the previous research question. Note that the differences are negative because they are calculated from the male point of view (men contacting women) but the sign does not play a role here because by definition, reciprocal ties are mutual and thus have no direction.

**Fig 2:**
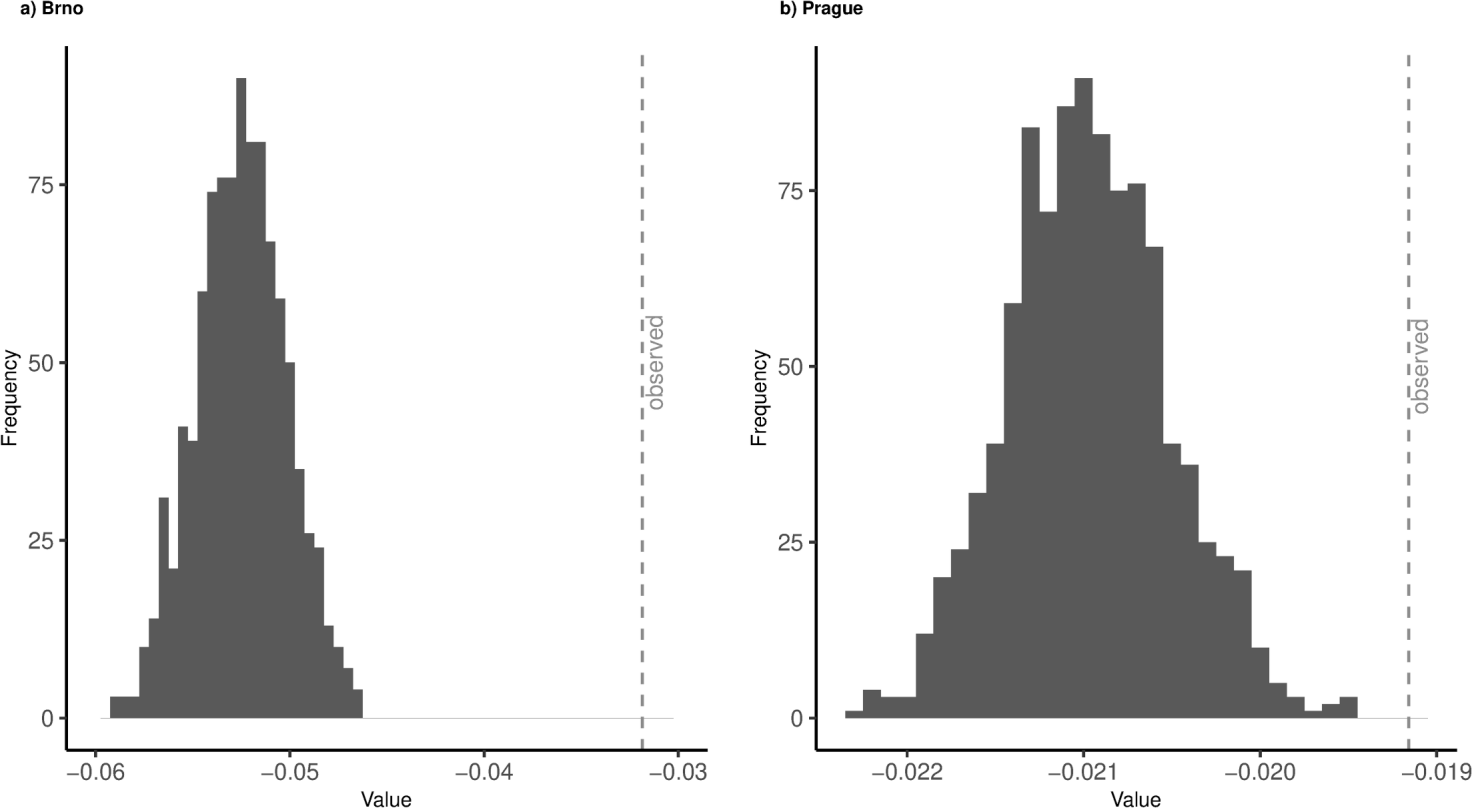
Simulated distributions of differences between mutually attracted users. Gray dotted line represents observed mean differences. Histograms represented simulated mean differences in 1,000 simulated networks.

In the Brno network, the observed difference in standardized indegrees is −0.032. This difference is considerably smaller than the difference in indegrees when men contact women, and closely resembles the difference between indegrees when women contact men in the network with all ties including non-reciprocated. In the Prague network, the average difference in standardized indegrees between is −0.019. Same as in the Brno network, this difference is much smaller than the difference in standardized indegrees when men contact women, and closely resembles the difference in indegrees when women contact men in the network with all ties including non-reciprocated ties. Compared to the observed difference, all the simulated networks for both cities display greater differences in mutually attracted users’ degrees when we condition on size and outdegree distribution (Fig 2).

We therefore respond to RQ4 by concluding the mutually attracted users are more homophilic in desirability compared to all swipes. This suggests that a lot of the swipes fail to develop beyond the initial attempt, and that the successful matches seem to reflect women’s preferences rather than men’s.

## Discussion

Our research corroborates previous studies regarding women’s higher desirability on online dating apps. Given the skewed gender ratio on the app, and the prevailing gendered dating norms, men were in the position of pursuers (sending more messages than receiving) and women in the position of “choosers” (receiving more messages than sending). Men pursued more desirable women than themselves, in line with the mechanisms of competition and non-reciprocity. We do not see the same pattern for women, who chose slightly less desirable partners on average. Here, our results differ from Bruch and Newman [12] who found aspirational pursuit for both men and women. However, women’s willingness to partner down should be taken with a grain of salt due to the low variability in men’s desirability.

Since pursuing mates and being successful in doing so are two inherently different things, we also looked at reciprocal matches. Even though the successful matches still display some differences on desirability, they are much more similar to the differences observed in swipes that women send to men than in swipes that men send to women. This is in line with Schaefer’s [31] concept of non-reciprocity – while the initial pursuits may be aspirational, through the process of rejection by more desirable counterparts, the successful matches tend to end up more homophilic.

### Limitations

The present study is not free from limitations. First, the analysis is based on a single dating app, which may suffer from selection bias. In general, dating apps are not representative of the general public, as they often include younger, more educated users from larger cities. In this case, the app was fairly new at the time of the data collection and was developed by two college students in a city with a major university. Thus, it is likely that it is biased towards more educated users who were early adopters of the app. Nevertheless, we believe that our study is a needed contribution to the literature on online dating and homophily. As all online dating studies share the same issue of being constrained to a specific online site, cumulative knowledge is the only way to assess whether the patterns they observe are truly happening or are merely results of the particular online dating platforms’ designs. Moreover, most studies concerning online dating currently rely on data from the US [e.g., 12,23,24,29,34,37] or Western Europe [41], a notable exception being Potarca and Mills [9]. Thus, we are not only replicating previous findings, we are also extending their geographical scope.

As with any digital trace data, the amount of information about the behavior of users is limited [43]. The specific app we used in this study did not record any information about the motivation of the users, yet these motivations may vary significantly among users from seeking one-night stands to searching for a life partner. Users interested solely in one-night stands may prioritize different characteristics (e.g., physical attractiveness) than those seeking long-term relationships [49,50]. This can, in turn, modify who ends up at the top or the bottom of the desirability hierarchy – but it would likely be a hierarchy nonetheless. However, disentangling short- and long-term relationship preferences in online dating is not straightforward, as many users report being open to both casual and long-term relationships, depending on whom they meet online. [51]. Similarly, the analysis is limited by the scope of the data as we only have a handful of information about users. Previous studies [29,52] controlled for factors such as education or religion. Since the app in our study did not record either of these pieces of information, we couldn’t control for it in our analyses. However, this information was also not accessible for users viewing the profiles and so it had no direct way of affecting their partner selection, yet we cannot rule out any more nuanced indirect effects. Furthermore, we cannot make any conclusions regarding users’ racial preferences given that Czechia is very strongly ethnically homogenous, with the vast majority of population being white Czechs [53].

Another limitation is that short-lived profiles could have influenced our results. Some of the users we classified as having low desirability, i.e., receiving few or no swipes, could have downloaded the app out of curiosity and quickly abandoned their profiles. Had the longevity of profiles been available for analysis, it could again be introduced as a control variable or more specifically, the temporal dynamics of the users’ usage of the app could be fully investigated in a longitudinal study. Longitudinal data could then in turn open an avenue for studying the adaptation of users’ strategies over time. This is potentially very important, as some studies suggest that users often update their profiles based on the “feedback” they receive from other users [35] and that dissimilar matches are more likely to dissolve with the continuing exchange between users [37].

### Future research

We only included heterosexual searches in the analysis, because of very low number of same-sex searches in the sample. While this is a limitation that we share with many previous studies utilizing digital trace data from online dating sites, it is also an opportunity for future research. The results based on the structure of heterosexual searches may substantially differ from same-sex searches, because in same-sex markets everyone can contact and be contacted by everyone else resulting in a one-mode directed network. In directed one-mode networks (which are some of the most frequently studied types of networks overall), a more complex network structure and more complex hierarchy among users can arise. For instance, there may be transitive triads of users in which one user only receives swipes from the two others, another receives and sends one swipe, whereas the last user only sends swipes, but receives none. This is a micro-scale hierarchy with the first user at the top and the last one at its bottom and it is manifested in directed one-mode network as a transitive triangle. Since this type of networks is one the basic ones, there are established measures and statistical models for these structures such as exponential random graph models [54] that can be readily applied to such data. Their application can in turn give us insights into the structure and dynamics of same-sex online dating markets. Another avenue for future research is the use of longitudinal data which would enable to see whether users of online dating apps change their behavior over time in association with the amount of swipes the receive. Relational event models [55] that enable modelling of time-stamped interactions could be a fruitful tool in this line of research.

Speaking of network methods and models, we initially attempted to use exponential random graph models for our data. Unfortunately, there are no currently available extensions for directed two-mode networks of these models and so we had to adjust their directed one-mode variants for our data by restricting the simulations that underlie their estimation procedures only to between-sex ties using structural zeros. However, this approach did not yield even remotely converged estimates despite substantially increasing the number of iterations and estimation runs. For this reason, we opted for conditional uniform graph tests to generate a baseline distribution of alternative networks wherein users could have chosen different receivers for their swipes. Similarly, we had to adjust standard topological measures to appropriately describe our networks and their structures. Future methodological research into both description and modelling of directed two-mode networks could greatly expand the analytical tools available for analysis of online dating networks.

In summary, our study shows that there is a hierarchy of users on online dating apps in terms of their desirability, with women being on top of this hierarchy. Furthermore, we provide evidence of men pursuing more desirable women, yet most of the mutually interested matches are more similar in terms of desirability. Our results highlight the usefulness of network perspective as it allowed us to characterize both the overall structure as well as the behavioral tendencies of users within it. Still, our results may be specific to one geographic context (Czechia) or to the specific dating app. In general, more quantitative studies using online dating data, spanning different geographical contexts, including non-Western countries, and including more diverse apps with different userbases (e.g., focusing on queer daters, long- or short- term relationships), algorithms, and functionalities, are needed. This in turn opens avenues for future research that may help us in increasing our knowledge of online dating and of online behavior in general.

## Supporting information

S1 Table Sensitivity analysis: Conditional uniform graph tests results. (PDF)
