# Supplementary material for: It takes two to tango: A directed two-mode network approach to desirability on a mobile dating app
Source: PLoS One. 2025 Jul 23;20(7):e0327477. doi: 10.1371/journal.pone.0327477 (PMC12286370; doi:10.1371/journal.pone.0327477)
Supplement: S1 Table — (PDF) [file pone.0327477.s001.pdf]

Supporting Information

It takes two to tango: A directed two-mode network approach to desirability on a mobile dating app

S1 Table reports the results of our sensitivity analysis. In this analysis, sent ties are weighted as the fraction of each sender's row total (outdegree) so that ties from highly active users weight less than those from less active ones. We then conducted the same CUG tests as described in the main text, section methods.

S1 Table. Sensitivity analysis: Conditional uniform graph tests results. Each row represents the given network of fractional edge values with its observed mean together with its simulated mean, standard deviations, and empirical p-values based on 1,000 permutations of the network.

		obs. mean	*Pr(<=obs. value)*	*Pr(>=obs. value)*	*sim. mean*	*sim. SD*
Brno						
	men contact women	-0.0172	0.00	1.00	-0.00512	0.0000330
	women contact men	0.00379	0.00	1.00	0.00633	0.0000750
Prague						
	men contact women	-0.00414	0.00	1.00	-0.000814	0.00000297
	women contact men	0.00157	0.00	1.00	0.00247	0.00000573
